# STAT4 gene polymorphisms in human diseases

**DOI:** 10.3389/fimmu.2024.1479418

**Published:** 2024-11-07

**Authors:** Yan Xia, Yanni Xie, Hao Zhang, Lunzhi Liu

**Affiliations:** ^1^ Hubei Provincial Key Laboratory of Occurrence and Intervention of Rheumatic Diseases, Minda Hospital of Hubei Minzu University, Hubei Minzu University, Enshi, Hubei, China; ^2^ Department of Nephrology, Minda Hospital Affiliated to Hubei Minzu University, Hubei Clinical Research Center for Kidney Disease, Hubei Minzu University, Enshi, Hubei, China; ^3^ Department of Endocrinology, Minda Hospital Affiliated to Hubei Minzu University, Hubei Clinical Research Center for Kidney Disease, Hubei Minzu University, Enshi, Hubei, China; ^4^ Laboratory of Immunology for Environment and Health, Shandong Analysis and Test Center, Qilu University of Technology (Shandong Academy of Sciences), Jinan, China

**Keywords:** STAT4, single nucleotide polymorphism, clinical significance, polymorphisms and therapeutic efficacy, autoimmune disease

## Abstract

Signal transducer and activator of transcription 4 (STAT4) is a member of the STAT family, which is a group of transcription factors that regulate cytokine signaling. Genetic polymorphisms in STAT4 strongly influence immune responses and disease outcomes, especially in cancer and autoimmune diseases. Several studies have indicated that certain STAT4 gene variants are associated with alterations in STAT4 expression and/or activity and that there is a close relationship between STAT4 polymorphisms and drug efficacy. However, the underlying mechanisms are complex, and the roles of these polymorphisms in disease acquisition, progression, and severity are of widespread concern. Therefore, we provide an overview of the clinical significance of polymorphisms in STAT4 and the mechanisms by which these STAT4 variants are involved in various diseases.

## 1 Introduction

STAT4 plays a central role in signal transduction, particularly in facilitating the production of biomolecules closely associated with autoimmune diseases. Studies have shown that mice lacking STAT4 exhibit a higher resistance to inflammatory conditions such as colitis, arthritis, diabetes, myocarditis, and experimental autoimmune encephalitis (1–6). In the human genetic system, the STAT4 gene is located on chromosome 2q32.2, with its DNA sequence spanning approximately 220 kilobases (kb) and containing 24 exons and 23 introns. The protein encoded by the STAT4 gene has a molecular weight of approximately 84 kDa and consists of 748 amino acids. Among its family members, the STAT4 protein exhibits the highest tissue specificity and is highly expressed in the lymph nodes, myeloid lineage, testicular tissue, and skin. Structurally, STAT4 resembles other STAT proteins in that it is composed of multiple conserved regions in both structure and function, including the N-terminal domain (NTD), the coiled-coil domain (CCD), the DNA-binding domain (DBD), a linker domain (LD), the Src-homology 2 (SH2) domain, and the C-terminal transcription activation domain (TAD) (7). Functionally, the active form of STAT4 is primarily activated through phosphorylation by Janus kinase 2 (JAK2) and tyrosine kinase 2 (TYK2), forming pSTAT4, which promotes STAT4 dimerization, nuclear translocation, and transcriptional activation of its target genes (8, 9). Notably, during this process, the SH2 domains of STAT4 proteins bind to tyrosine-phosphorylation sites (pYs) on receptor complexes as well as pYs on other STAT proteins and work together with the NTD to mediate the formation of homodimers or heterodimers by STAT4. Notably, studies indicate that STATs lacking the NTD cannot form tetramers at DNA-binding sites (10), which potentially impedes the full transcriptional activation of many STAT target genes (11). The CCD and TAD domains allow the STAT4 protein to bind to other transcription factors or co-activators, and depending on the presence of the TAD domain, there can be two spliced variants of STAT4: STAT4α, which contains the TAD, and STAT4β, which lacks it (12).

Although both STAT4α and STAT4β can activate transcription in primary cells and cell lines, studies have demonstrated that the absence of the TAD domain in STAT4β can markedly alter its function (12). In addition, studies have indicated that genetic polymorphisms in STAT4 also influence immune responses and disease susceptibility. For example, unstimulated cells transfected with STAT4 H623Y or A635V variants exhibited greater accumulation of STAT4 in the nucleus (13). Therefore, this review aims to summarize various reports on STAT4 polymorphisms, the effects of these polymorphisms on disease susceptibility and treatment effectiveness, and the mechanisms through which these STAT4 variants are involved in these diseases.

## 2 Biological effects of STAT4

**Figure 1:**
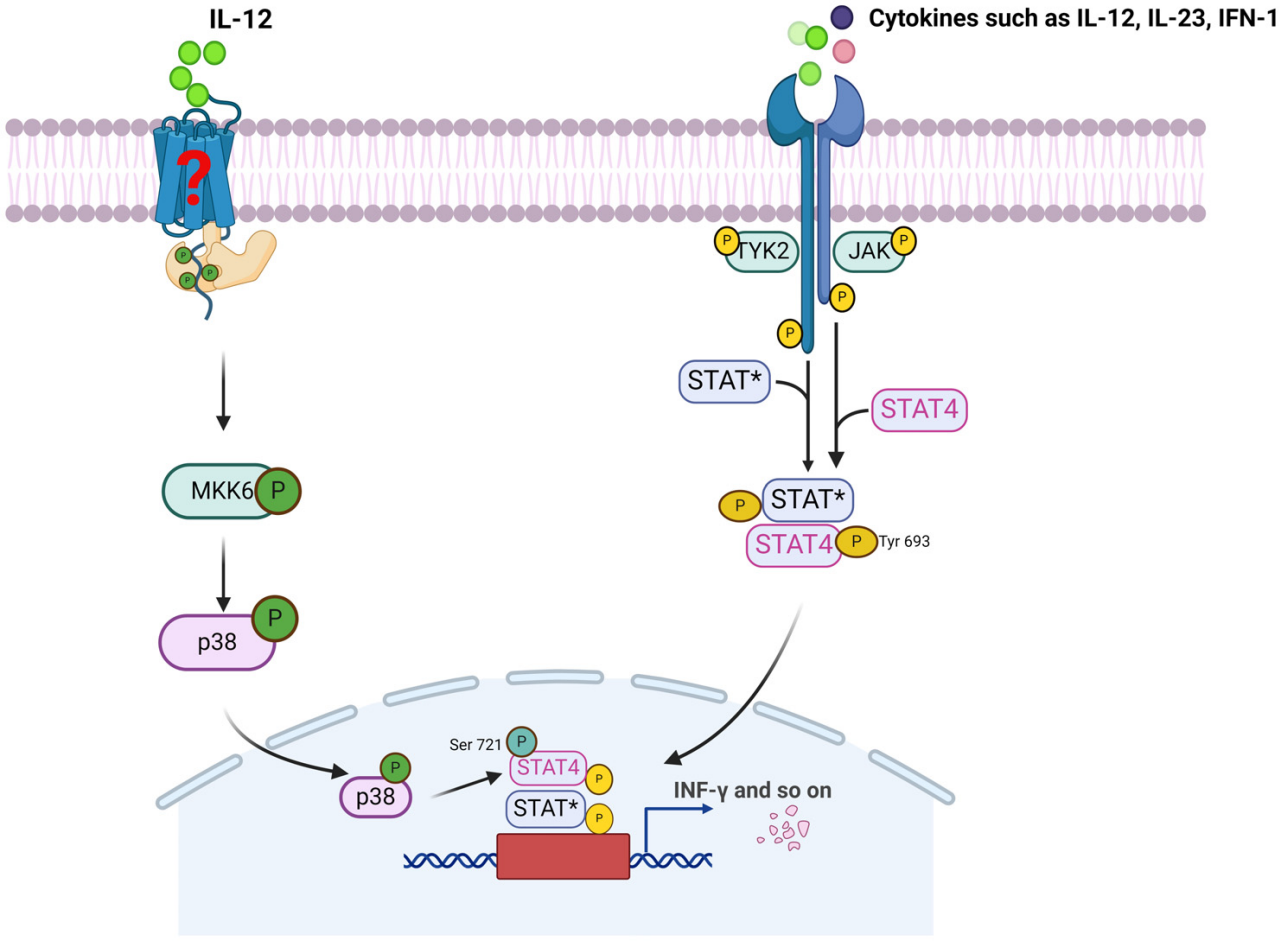
The activation of STAT4 and its mainly signaling pathway. JAK, Janus kinase; TYK2, Tyrosine kinase 2; STAT*, Signal transducer and activator of transcription 4 or other STAT; MKK6, MAP kinase kinase 6.

As shown in **Figure 1**, STAT4 is mainly activated within the cytoplasm by cytokines such as IL-12, IL-23, and type I interferons, via the JAK2 and TYK2 kinase pathways and/or the p38/MKK6 signaling cascade. This, in turn, leads to the phosphorylation of STAT4 on tyrosine and/or serine residues, thereby promotes STAT4 dimerization, nuclear translocation. Ultimately, this leads to the transcriptional activation of STAT4’s target genes, fulfilling its functional role. Studies have indicated that JAK activation most likely results in the phosphorylation of tyrosine residue 693 on STAT4, which facilitates STAT4 dimerization and nuclear translocation. Mutation at this site completely abrogates IL-12-induced STAT4 transcriptional activity. In contrast, activation of the p38/MKK6 signaling pathway leads to phosphorylation of STAT4 at serine residue 721. Unlike tyrosine phosphorylation, it has been proposed serine phosphorylation of STAT4 is dispensable for nuclear translocation or DNA binding of STAT4, but is required for STAT4 full transcriptional activation (14). Furthermore, it was suggested that serine phosphorylation of STAT4 is partially dependent on precedent tyrosine phosphorylation of STAT4, whereas tyrosine phosphorylation of STAT4 can be seen even in the absence of serine phosphorylation (15), which further implying that the phosphorylation of STAT4 by p38 occurs in the nucleus. Additionally, the phosphorylation of residues serine 733, 743, and 713 is also closely associated with the activation of STAT4. It is speculated that these non-canonical serine phosphorylations may cause the dissociation of STAT4 from the Leukemia Inhibitory Factor Receptor (LIFR), enabling its subsequent nuclear translocation (16).

Although numerous immune cells do not express STAT4 in their basal state (17, 18), the regulation of STAT4 expression appears to play a crucial role in the immune response. For example, STAT4 expression is rarely detected in immature DCs (iDCs) and mucosal mast cells (MMCs), while highly expressed in matured DCs, connective tissue-type mast cells (CTMCs) and activated monocytes (19, 20). Besides, Stat4 expression in T cells is greatly influenced by their state of activation. Human peripheral blood T cells do not have Stat4 in their basal state, but its expression is markedly induced following stimulation (18). It is worth noting that there are differences in the activation mechanisms of STAT4 among different species. For example, in mice, STAT4 can only be activated by IL-12, whereas in humans, STAT4 is not only activated by IL-12 but also phosphorylated by IFN-α (21, 22). Moreover, the activators of STAT4 may vary among different cell types, with monocyte-expressed STAT4 responding to IFN-α but not IL-12 (17).

**Figure 2:**
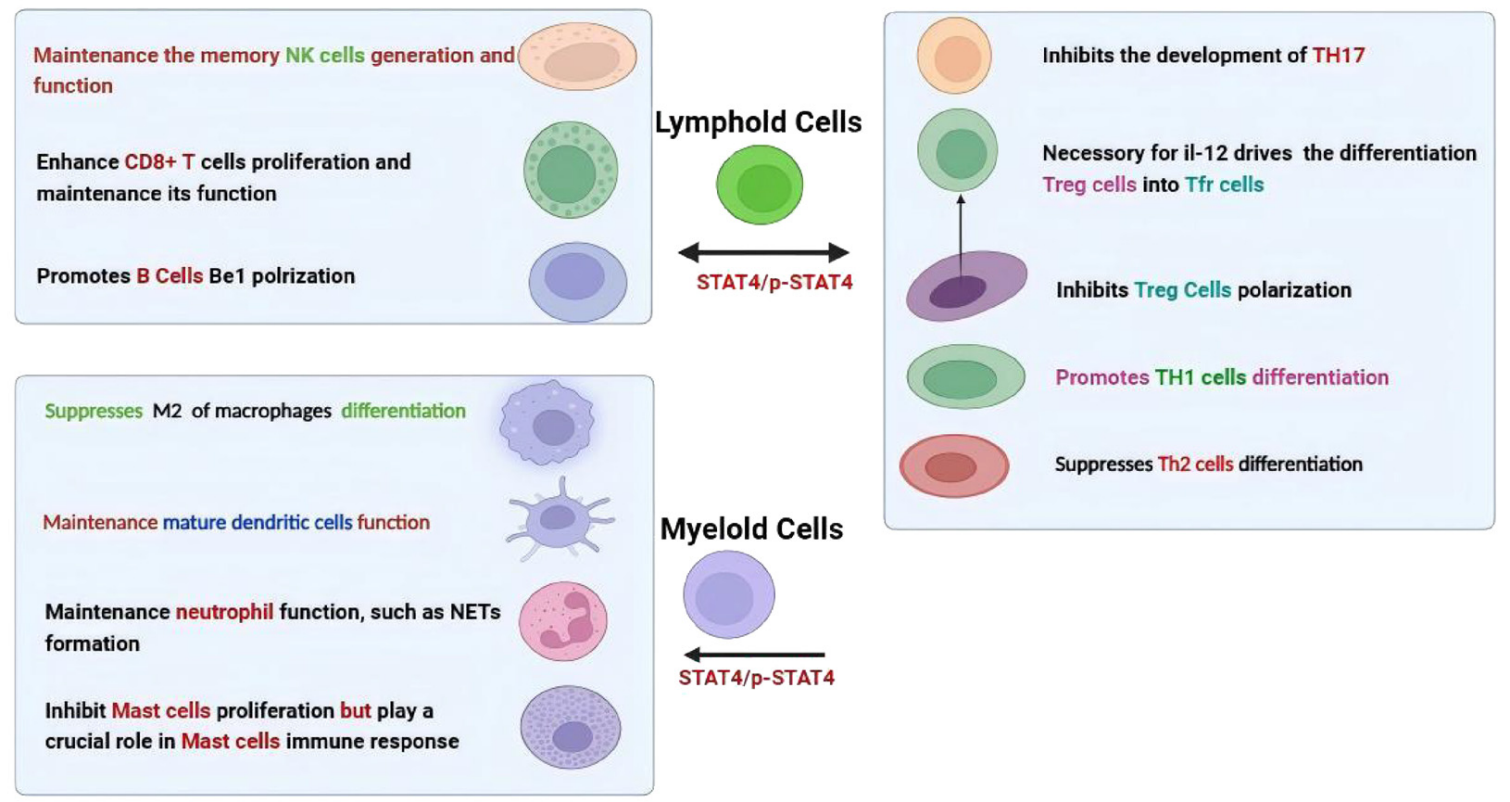
Divergent roles of STAT4/p-STAT4 in different immune cells.

As shown in **Figure 2**, the function of STAT4 in the immune system mainly encompasses both innate and adaptive immune responses. In particular, for T helper (Th) cells, STAT4 is vital in regulating their development and effector functions. It was suggested STAT4 promotes the differentiation of Th1 cells mainly through upregulating the expression of interleukin-12 receptor β2 (IL-12Rβ2) and enhancing IL-12Rβ2-mediated signaling (23–25). STAT4 is also necessary for follicular helper T (Tfh) cell development, and increased activation of STAT4 in these cells leads to abnormal production of IL-21 and IFN-γ (26, 27). Moreover, STAT4 is involved in the process by which IL-12 drives the differentiation of human regulatory T (Treg) cells into T follicular regulatory (Tfr) cells (28). In contrast, CD4+ T cells lacking STAT4 have a greater tendency to differentiate into Th2 cells (3, 29), and STAT4 also demonstrates a potent ability to inhibit the development of Th17 precursor cells by inducing their conversion into IFN-γ-producing cells (30). In terms of CD8+ T cells, STAT4 plays a vital role in the homeostatic self-renewal of CD8+ T cells and IL-12–induced STAT4 is crucial for the proliferation of effector CD8^+^ T cells in an mTOR-dependent manner, and upregulated STAT4 expression significantly enhances their survival capacity and infiltration (31–34). Conversely, its absence impairs its effector functions (32, 35). Furthermore, the activation of natural killer (NK) cells also strictly depends on STAT4, which influences NK cell function by regulating the production of IFN-γ and perforin (36–38). High levels of STAT4 contribute to the maintenance of memory NK cell generation, as the number of NK cells in wild-type (WT) mice is greater compared to those in STAT4-deficient mice after infections with mouse cytomegalovirus (MCMV) (39). Meanwhile, pSTAT4 is necessary for the Be1 polarization of human naive B cells (40).

While, STAT4 not only actively participates in the differentiation of lymphocytes but also plays a key role in the activation of myeloid cells such as monocytes and dendritic cells. For instance, studies have shown that the alternative activation (M2) of macrophages is enhanced in diet-induced obese mice due to the lack of STAT4 (41). STAT4 is required for intrinsic signaling in mature dendritic cells (mDCs) function (42), and STAT4-deficient plasmacytoid dendritic cells (pDCs) exhibit defects in the production of IFN-γ (43). Contradictorily, despite research indicating that STAT4 regulates IL-6 through an autocrine mechanism to inhibit the proliferation of CTMCs, it plays a crucial role in mast cell immune response (20, 44, 45). Furthermore, recent research has shown that neutrophil-specific deletion of STAT4 resulted in enhanced susceptibility to methicillin-resistant Staphylococcus aureus (MRSA) (46). This discovery indicates that STAT4 also plays a crucial role in neutrophil function. Specifically, it has been found to exert important effects through multiple aspects such as the production of reactive oxygen species (ROS), chemotaxis, and the formation of neutrophil extracellular traps (NETs).

## 3 Polymorphisms in the *STAT4* gene

**Figure 3:**
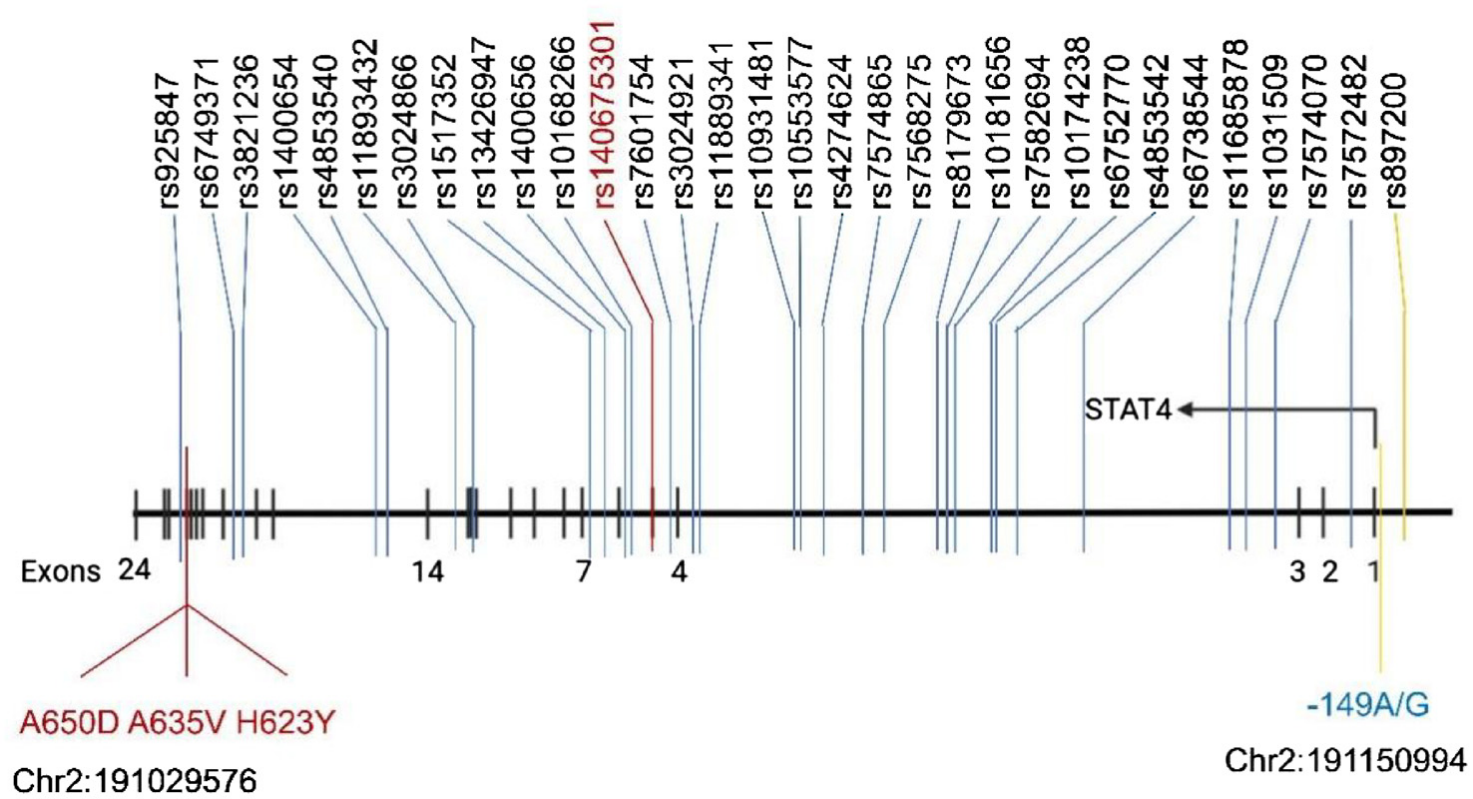
Reported polymorphisms (SNPs) in the STAT4 gene associated with disease occurrence.

Various polymorphisms within and surrounding the STAT4 gene sequence have been identified. These variations can be broadly categorized into three classes: those in intron regions, those in the 5′-flanking DNA, and those within the open reading frame. Currently, STAT4 polymorphisms and their potential links to diseases have been reported in more than one hundred studies from various countries. These are illustrated in **Figure 3**.

### 3.1 Single nucleotide polymorphisms in introns

Presently, the majority of known *STAT4* gene variations occur within introns. During RNA processing, the intron sequences of the primary RNA transcripts derived from both normal and variant alleles are excised, resulting in the production of identical STAT4 mRNAs and subsequent synthesis of identical STAT4 proteins. Notably, however, SNPs within the introns of *STAT4*, particularly the rs7574865 polymorphism in intron 3, play an important role in regulating STAT4 expression. For example, studies have indicated that patients with systemic lupus erythematosus (SLE) carrying the rs7574865 T allele have increased levels of STAT4 mRNA in osteoblasts and peripheral blood mononuclear cells (PBMCs) (47, 48), and when T cells from these patients are stimulated with IL-12 and IFN-α, the levels of STAT4 protein and its phosphorylated form, pSTAT4, are markedly increased (49). Besides, mutations within the *STAT4* third intron contain many elements that may alter CD4+ T cell activation induced by IL-12, which may affect the rates of *STAT4* transcription and consequently IFNγ production in CD4+ T cells (50). In addition, research has revealed a significant correlation between this risk allele and the methylation status at position -172 of the STAT4 promoter (51). In addition, the SNPs rs3821236, located in intron 16, and rs3024866, located in intron 13, have also been correlated with increased STAT4 expression (48). However, the effects of genetic variants appear to be context-specific, such as significantly elevated STAT4 levels in the serum and peritumoral tissue of hepatocellular carcinoma (HCC) patients with the rs7574865 GG genotype (52). Furthermore, research suggests that rs4853540 located in the STAT4 gene may map to an enhancer of STAT1 (53). And there exists a genotype-dependent repressive element in the DNA surrounding rs11889341. The risk allele rs11889341C enhances HMGA1 binding capability, resulting in reduced repressor activity and consequently higher levels of STAT1 expression (54).

### 3.2 Single nucleotide polymorphisms in 5′-flanking DNA

A second type of allelic variation occurs within the 5’-flanking region of the *STAT4* promoter. Shin HJ and his colleagues demonstrated evidence for allelic variation (−149A/G) in noncoding exon 1 of the STAT4 gene, which is located in the 5’-flanking region of the essential promoter. Subsequently, direct sequencing analysis revealed that this noncore promoter region allelic variation frequently appeared in patients with rheumatoid arthritis (RA) and asthma. However, luciferase reporter gene assays of genes transcriptionally controlled by either the wild-type STAT4 promoter or the variant promoter revealed that this polymorphism had no significant effect on promoter activity (55).

In addition, another SNP located at the edge of the 5’ promoter region of STAT4 (rs897200) has been identified (56). Notably, through analysis using the Regulomedb database and predictions made with ALGGEN PROMO software (57), it was discovered that the site at which this SNP occurs could potentially serve as a transcription factor-binding site. In line with this, subjects with the AA genotype presented significantly higher STAT4 mRNA levels in PBMCs and skin cells than did subjects with the GG genotype, and luciferase activity was notably increased in cells harboring the A allele (58).

### 3.3 Single nucleotide polymorphisms in extrons

A third type of allelic variation has been identified in exons within the STAT4 open reading frame. This type of polymorphism has the potential to affect both protein structure and function, which is different from the effects of reported STAT4 polymorphisms in noncoding sequences. Recently, several SNPs have been identified in exons.

Through a genome-wide association study (GWAS), Saevarsdottir S and colleagues reported that a rare missense variant in *STAT4*, rs140675301-A, causes a 2.27-fold increase in the risk of seropositive RA. Specifically, this genetic variant causes the replacement of hydrophilic glutamic acid with hydrophobic valine (Glu128Val) in a conserved surface-exposed loop between the N-terminal domain and the helical coiled coil domain (59), although the direct impact of the mutation at this site on protein function remains unknown.

Remarkably, three other heterozygous polymorphisms (*STAT4* c.1904 C→T, c.1949C→A, and c.1867C→T), resulting in A635V, A650D, and H623Y amino acid substitutions, respectively, in a specific region of the gene that encodes *STAT4* were identified. All of these polymorphisms, occur in the region of the STAT4 gene that encodes the SH2 domain. They also discovered that, in unstimulated cells containing STAT4 variants, there was greater accumulation of STAT4 in the nucleus, and the levels of pSTAT4 were increased, and compared with those in control cells, the levels of pSTAT4 not only increased but also remained elevated for a comparatively longer duration in the interferon-stimulated cells. In addition, they found that mutant *STAT4* dimers are more stable than wild-type *STAT4* dimers (13), but little is known about how these mutations promote the phosphorylation of STAT4 and maintain the stability of its dimers. Regardless, this experiment further suggested that the SH2 domain is strongly linked to the activity of STAT4, indicating that mutations within this domain could significantly alter the phosphorylation and dimerization of STAT4, which in turn affects its transcriptional activity and potentially contributes to the occurrence of disease.

## 4 Genetic variation and disease susceptibility

STAT4 can be activated by distinct types of cytokines in multiple cells via the JAK-STAT pathway or p38/MKK6 pathway. Subsequently, it acts as a transcription factor to regulate the expression of various genes. This pivotal function makes STAT4 a central mediator in the induction of inflammation during protective immune responses and immune-mediated diseases. Importantly, numerous studies have identified the association between STAT4 polymorphisms and disease susceptibility.

### 4.1 Polymorphisms and cancer

Associations between STAT4 SNPs and cancer susceptibility have been reported in different cancer types, but the results have been inconsistent. The STAT4 polymorphism rs7574865 has been extensively studied in the context of hepatocellular carcinoma (HCC) and chronic hepatitis B virus (HBV) infection. Although it is widely accepted that the C allele of rs7574865 increases the risk of HCC and is strongly associated with HBV-related HCC, especially in Asian populations (60–62). However, several studies, such as that of Chen and colleagues, have failed to replicate these findings (63). Additionally, a recent report indicated that this SNP (rs7574865) does not influence the risk of developing HCC in Latin American or European populations (64), suggesting that the association between rs7574865 and HCC risk may be specific to certain populations, and more studies are needed. In addition, a recent study suggested a potential association between STAT4 rs11889341 or rs10174238 and HCC risk among the Chinese Han population (65).

Additionally, the variants of *STAT4* rs7574865T and rs1400656G serve as protective alleles against the risk of lung cancer (66). *STAT4* rs6738544A was significantly associated with pancreatic cancer risk (67). Furthermore, logistic regression analysis revealed that rs4274624 (*STAT4*) is linked to an elevated risk of breast cancer, whereas STAT4 rs925847 is associated with a reduced risk of breast cancer (68).

### 4.2 Polymorphisms and autoimmune diseases

SNPs in STAT4 have been reported as risk factors for the development of autoimmune diseases, including RA, SLE, lupus nephritis (LN), type 1 diabetes (T1D), psoriasis, inflammatory bowel disease (IBD), Behçet’s disease (BD), Sjögren’s syndrome (SS), systemic sclerosis (SSc), primary biliary cirrhosis (PBC), and other diseases (**Table 1**).

**Table 1:** Association between STAT4 SNPs and autoimmune disease.

Disease	Identifier or genotype of SNPs(risk)	Sample population	Reference
RA	rs7574865(T)	Patients vs Control, In North America/Africa//Asian/Polish Population/South America/European populations	(69–77, 81, 83)
	rs10181656(G) rs11889341(T)rs7574865(T), rs8179673(C),	In Asia	(78, 79)
	rs11889341(C)/rs7574865(T)	Patients with RF-positive RA vs patients with RF-negative RA	(80, 81)
	rs140675301(A)	Seropositive RA vs Seronegative RA	(59)
	rs1018165(G)/rs7574865(T)	ACPA-positive RA vs ACPA-negative RA	(81, 84)
SLE/LN	rs7574865(T)	In Asian/European populations/North America/South East Asian	(85–87)
	rs10181656(G)	multiple racial groups	(92, 96)
	rs7582694(C)	Asian/European population	(92, 94–96)
	rs11889341 (T)	Asian	(98, 100)
	rs7601754 (T)	Asian/European ancestry	(91, 97)
	rs10168266 (T)	South East Asian/multiple racial groups	(90, 92, 93, 98)
	TTT haplotype (rs10168266/rs11889341/rs7574865)	In Asian	(98)
IBDs	rs7574865 (T)	In all population	(104)
Type 1 diabetes	rs7574865 (T)	European/Africa	(107–110)
	rs11889341 (T), rs7574865 (T), rs8179673 (C), rs10181656 (G)	In Asian; the early-onset subgroup (less than 7.6 years old)	(111)
	rs11889341 (T)	In Asian (weak association)	(111)

#### 4.2.1 RA

Over the past two decades, the potential effects of STAT4 SNPs on susceptibility to RA have been evaluated. Among these, STAT4 rs7574865 is the most well studied and has been identified as an important risk factor for RA across multiple ethnic group (69–77). Specifically, the T allele of rs7574865 is associated with an increased risk of RA, with the TT genotype predominantly found in RA patient. Besides, studies have also revealed significant associations among the SNPs rs10181656(G), rs11889341(T), rs7574865(T), and rs8179673(C) in the *STAT4* gene and RA (78, 79).

Furthermore, some research findings indicate that STAT4 gene polymorphisms may be involved in regulating the seropositive status of RA patients. Specifically, researchers have reported that the frequency of the T allele and TT genotype of rs11889341 is significantly lower in the rheumatoid factor (RF)-positive subgroup of RA patients than in the RF-negative subgroup, but there is a lack of direct evidence linking rs11889341 with RA risk (80). Additionally, the frequency of RF positivity was significantly greater among RA patients with the rs7574865 T allele (81). Furthermore, the rs7574865 and rs10181656 loci in the STAT4 gene were associated with anti-cyclic citrullinated peptide (ACPA)-positive RA (81–84), and a rare missense variant in the exon of STAT4, rs140675301A, has also been shown to increase the risk of seropositive RA (59).

#### 4.2.2 SLE and LN

The association between STAT4 gene polymorphisms and SLE/LN remains controversial according to published studies. However, several meta-analyses have consistently indicated that the T allele or TT genotype of the STAT4 rs7574865 polymorphism serves as a risk factor for SLE across different ethnic populations, despite variations in prevalence rates among different ethnicities (85–87). Moreover, higher levels of IFN-γ (interferon-γ) have been reported in TT allele carriers (88). Interestingly, there are significant differences in the functional manifestations of the STAT4 rs7574865 variant between healthy individuals and patients with SLE, and its function may also be influenced by other mutation sites. Specifically, the STAT4 risk allele rs7574865 T has contrasting effects on cells from healthy individuals compared with those from patients with SLE. In SLE patients carrying the STAT4 risk allele rs7574865[T], T cells exhibit increased STAT4 protein and pSTAT4 lever, and elevated IFN-γ production after PHA/interleukin (IL)-2 activation, whereas in healthy individuals, STAT4 risk allele carriers have reduced pSTAT4 levels in CD8+ and CD4+ T cells (89). However, CD4+ naive T cells from both healthy individuals and SLE patients carrying the non-risk homozygous (NR/NR) genotype (rs7582694G and rs7574865G) exhibit significantly lower levels of STAT4 compared to cells carrying the high-risk homozygous (R/R) genotype (rs7582694C and rs7574865T) during T-cell differentiation, and activation of cells from healthy individuals and SLE patients with the R/R genotype shows increased levels of transcriptionally active STAT4 and production of interferon-γ (50).

In addition, several studies have suggested that the minor allele polymorphisms rs10168266T, rs7601754T, rs7582694C and rs3821236A are also associated with SLE (48, 84, 87, 90–97), and that the TTT haplotype (rs10168266/rs11889341/rs7574865) is also linked to SLE (98). Moreover, SLE-smoking patients with the STAT4 SNP rs11889341T allele have a significantly increased risk of LN (99), and also SLE patients with the rs7582694C and rs7574865T allele exhibit a significantly increased incidence of severe renal insufficiency (100, 101).

#### 4.2.3 IBDs

To date, the associations between STAT4 variants and IBD, specifically ulcerative colitis (UC) and Crohn’s disease (CD), remain uncertain. Previous meta-analyses have suggested that the STAT4 rs7574865 T allele may confer increased susceptibility to UC (102, 103). Subsequently, another study analysis has further clarified that this genetic polymorphism is most strongly associated with UC susceptibility among Caucasians (104). However, a case-control association study conducted in a Korean population revealed that, while the SNP rs925847 polymorphism provides a protective effect against UC, none of the other tested STAT4 SNPs (including rs11889341, rs8179673, rs6752770, rs10168266, rs10181656, and rs11685878) were linked to UC susceptibility (105).

In addition, a protective role for rs7568275T and rs10174238 against the risk of Crohn’s disease (CD) was suggested (106). However, a final meta-analysis found no significant association between STAT4 polymorphisms and CD susceptibility (104).

#### 4.2.4 T1D

Although it has been proven previously that several SNPs in the STAT4 gene contribute to the genetic predisposition to T1D, the role of STAT4 polymorphisms in T1D is poorly understood. This association was first observed in a genetically homogeneous population, where susceptibility to type 1 diabetes was associated with a significant increase in the frequency of the STAT4 rs7574865 T allele (107). Subsequently, several studies on Asian, African and European populations confirmed that rs7574865 plays a role in the incidence of T1D (102, 108, 109), and that carriers of the rs7574865 minor T allele presented earlier T1D onset (110). In addition, analysis of the early-onset subgroup revealed that rs11889341, rs7574865, rs8179673, and rs10181656 were significantly associated with susceptibility to T1D, whereas only a weak association was observed between rs11889341 and T1D in general (111). Over all, these findings suggest that certain STAT4 SNPs may be particularly relevant in the context of early-onset T1D.

#### 4.2.5 SSc

The results of studies on the role of STAT4 SNPs in SSc are somewhat conflicting. Similar to other autoimmune diseases, the T allele of rs7574865 has been identified as a susceptibility factor for SSc (112), and a meta-analysis conducted by Xu et al. encompassing six studies revealed that, depending on the extent of skin involvement, the frequency of the STAT4 (rs7574865) risk T allele was increased in both limited cutaneous (lcSSc) and diffuse cutaneous (dcSSc) SSc patients compared with healthy individuals (113). This finding indicates a potential subtype-specific association, but more experiments are needed to verify this observation. Other variants of STAT4, such as rs11889341A and rs10168266T, have been implicated in systemic sclerosis (SSc) in various studies (114, 115). Moreover, carriers of the rs3821236 A-allele and rs7574865 T-allele, along with rs10168266 T-allele carriers, exhibited an increased prevalence of pulmonary fibrosis in SSc patients, and carriers of rs7574865 and rs10168266 T-alleles were also strongly associated with the presence of anti-topoisomerase I (ATA) (115).

#### 4.2.6 Behçet’s disease/periodic fever, aphthous stomatitis, pharyngitis, and cervical adenitis

GWASs in patients with BD have been performed in Turkish, Japanese, Chinese, and Iranian populations, and *STAT4* polymorphisms is considered a common risk factor for BD (58, 116–118). Specifically, the rs7572482 risk allele T, rs7574070 risk allele A, and rs897200 risk allele A have been found to be associated with BD. In addition, studies have explored the relationship between gene polymorphisms at other loci of STAT4 and BD, and revealed that the GG genotype of rs7574865 may be a risk factor for BD patients (119, 120). However, a study conducted in the Korean population revealed no association between SNPs of STAT4 (including rs7574070, rs1031508, rs897200, and rs7572482) and the risk of BD in an analysis of individual polymorphisms (121).

In addition, studies have revealed that PFAPA syndrome shares genetic similarities with BD, and that the rs7574070 risk allele A is also an important susceptibility locus for PFAPA (122).

#### 4.2.7 SS

Several studies have been conducted to determine the associations between *STAT4 polymorphisms* and susceptibility to SS. Among Europeans, a statistically significant association between SS and STAT4 variants was observed, especially the insertion−deletion polymorphism rs10553577 located in the third intron, which exhibited a particularly strong association (123). Additionally, the T allele of rs7574865 at the genetic locus is significantly more common in SS patients, and carriers of this allele exhibit a higher risk of monoclonal component and leukopenia (124). Consistent with these findings, other GWAS analyses also established associations between SS and the gene regions of STAT4, including rs11889341, rs8179673 (125). Moreover, research indicates that rs7574865, rs7582694, and rs10168266 are significantly associated with primary Sjögren’s syndrome (126–130).

#### 4.2.8 Juvenile idiopathic arthritis

Studies have suggested that the rs7574865 risk allele T plays a role in the development of JIA and is associated with this condition in Han Chinese and American populations (131, 132), but not in Iranian or Greek populations (133, 134). Additionally, in Han Chinese populations, the G allele of rs11893432 was notably associated with an increased risk of oligoarticular JIA, whereas the A allele of rs10931481 and the C allele of rs1018981 were suggested to be associated with higher risk of polyarticular JIA (135).

#### 4.2.9 Optic neuritis/neuromyelitis optica spectrum disorder/idiopathic inflammatory myopathy

Additionally, STAT4 polymorphisms have been reported to be associated with other diseases. For example, a recent study revealed that both the G-G-A-C and C-T-A-T haplotypes of STAT4 (rs10181656, rs7574865, rs7601754, and rs10168266) are associated with the occurrence of optic neuritis (136). Besides, four STAT4 variants, including rs7574865 T, rs10181656 G, rs13426947 A, and rs10168266 T, have been reported to be associated with an increased risk of NMOSD, and similar to SLE, the G allele of rs7601754 also displays a protective effect against NMOSD (137). In addition, studies have revealed an association between STAT4 variants and IIM. Through candidate gene studies, it was discovered that both polymyositis and dermatomyositis are strongly associated with the rs7574865T allele (138). Additionally, by constructing a regional association plot of the STAT4 locus, two other SNPs, rs4853540 and rs6752770, were identified as having a significant association with IIM (53). Although these loci do not overlap, these studies collectively reinforce the association between STAT4 and IIM.

### 4.3 Infections disease

Several studies have indicated an association between STAT4 polymorphisms and infectious diseases. It was well known the C allele of rs7574865 may enhance susceptibility to hepatitis B virus (HBV) infection (61, 139). In addition, the impact of STAT4 polymorphisms appears to be more pronounced in young patients with pulmonary tuberculosis (PTB). A study revealed an association between STAT4 SNPs (rs6752770, rs3024861, rs7572482, rs1031509, rs1400654 and rs897200) and PTB, particularly highlighting a strong correlation between rs897200 and younger PTB patients (pulmonary tuberculosis onset <25 years) (56). While another study revealed that the rs4853542A allele reduces the risk of tuberculosis in younger adults after applying Bonferroni correction (140). Moreover, the rs7574865 TT genotype was identified as a risk factor for cytomegalovirus (CMV) infection (141).

### 4.4 Other diseases

In addition, the G allele of rs1031509 is significantly associated with an increased risk of developing doctor-diagnosed asthma when induced by exposure to benzo[α]pyrene in the environment (142), and SLE patients carrying the rs11889341 T risk allele appear to have an increased risk of myocardial infarction (MI) and nephritis (99). Moreover, an association between the rs7574865T allele and type-1 autoimmune hepatitis was observed (143), and an analysis of drug-induced liver injury patients revealed a trend toward an association between a STAT4 variant allele (rs7574865T) and hepatocellular injury, although this association was not significant (144).

Overall, these findings suggest that STAT4 polymorphisms have clinical significance. However, more studies with larger samples are needed to verify the roles of STAT4 polymorphisms in these diseases.

## 5 Polymorphisms and therapeutic efficacy

Clinical evidence suggests that genetic variations may interfere with the mechanism of drug action. Several studies have reported that SNPs of STAT4 are associated with the clinical efficacy of tumor necrosis factor (TNF) inhibitors in the treatment of RA patients (145), and the STAT4 rs7574865 T allele is associated with the absence of a good/moderate EULAR response at 2 years of treatment in RA patients and ETN-treated patients (146). In addition, carriers of the risk allele exhibit exaggerated CD4+ T-cell activation that, in the context of SLE, contribute to more severe disease, and R/R patients may benefit from blockade of the IL-12/STAT4 pathway (50). Additionally, a prospective cohort study suggested that the effectiveness of peginterferon-α (PEG-IFN) therapy can be altered by STAT4 polymorphisms. Specifically, patients in the GT/TT group presented a notably higher HBeAg seroconversion rate and hepatitis B surface antigen loss rate than those in the GG group (147). Similarly, another study revealed that the SNP rs757486 TT genotype was associated with a greater virological response to PEG-IFN therapy, regardless of baseline HBeAg status (148). However, importantly, this correlation was not observed in nucleoside (acid) analog treatments (149).

In addition, a recent study implicated STAT4 gene polymorphisms as the primary genetic factors that play a role in DPM disease, and the JAK inhibitor ruxolitinib has shown promising results in reducing inflammation in patients with DPM caused by gain-of-function variants in STAT4 by precisely targeting the STAT4 signaling pathway (13). Therefore, STAT4 genetic variation can significantly impact drug treatment effectiveness, emphasizing the importance of understanding these variations for the advancement of personalized medicine and improved patient outcomes.

## 6 Conclusion

Although STAT4 functions as an immunoregulator contributing to diverse human diseases, it is merely one element within a complex network of both proinflammatory and anti-inflammatory molecules. Its activation is tightly regulated by various cytokines, such as interleukin-12, interferon-gamma, and interleukin-1. Interestingly, the effects of STAT4 depend not only on its absolute expression but also in its functional state. The activity of STAT4 can be modulated by regulatory factors such as suppressor of cytokine signaling protein (SOCS) (150, 151), which inhibits its activity by blocking its receptor binding (152).

The majority of STAT4 gene polymorphisms described here occur within introns, and genetic editing has confirmed that the most significantly associated SNPs linked to autoimmune disease are located in the third intron of the gene (192, 153, 154). Among them, three single nucleotide variations, rs7574865 G/T, rs7582694G/C, and rs10181656C/G, are particularly associated with autoimmune disease, and these three loci are in a state of strong linkage disequilibrium. Specifically, the rs7574865 G > T mutation has been extensively studied, with numerous reports indicating that patients who possess a T allele have an increased risk of autoimmune diseases such as RA, multiple sclerosis, SLE, and primary SS (49, 102, 155). However, this situation is reversed in the context of cancer. Specifically, the G allele of rs7574865 has emerged as a risk factor for cancer development and progression, and STAT4 levels in serum and peritumoral tissue of HCC patients with the GG genotype are significantly higher than those found in TT or TG carriers (52, 139). This highlights the complexity of STAT4 gene variations and their context-dependent effects on different diseases.

Further evidence has indicated associations with autoimmune diseases beyond the SNP variants in intron 3. Specifically, the intronic region spanning from intron 14 to exon 17 (encompassing rs3821236G/A, rs3024886C/T, and rs3024877G/A), as well as the region extending from exon 4 to intron 14 (including rs10168266C/T, rs1517352C/A, rs13017460G/A, and rs16833249T/C), has also demonstrated a significant correlation with autoimmune diseases, especially SLE (48). Specifically, rs3821236A and rs10168266T were significantly more common in patients with SLE than in healthy controls (98), and rs7574865 did not exhibit strong linkage disequilibrium with rs3024866 (R2 = 0.29) or rs3821236 (R2 = 0.42) (48). Furthermore, data from the HapMap project revealed significant differences in the frequency distribution and linkage relationships among STAT4 SNPs between Eastern and Western populations. This finding suggests that different variations may serve as independent risk factors for disease susceptibility, with some having stronger effects in specific populations.

Furthermore, given the intimate connection between STAT4 activity and its protein structure, genetic polymorphisms situated within exon region are likely to exert a more crucial influence on the progression of diseases. However, despite the intricate genetic regulation of STAT4 and its pivotal role in immune responses, the impact of genetic variations on disease susceptibility and outcomes is not absolute, as could be expected. Nonetheless, genetic variations influencing STAT4 activity clearly hold significance. Individuals endowed with a genetically predisposed capacity for heightened STAT4 activation may experience more pronounced inflammatory reactions under certain circumstances. Therefore, more research is needed to elucidate the associations between STAT4 polymorphisms and diseases to further clarify how different polymorphisms affect the function of STAT4 and how these effects are related to the pathogenesis and progression of various diseases.
